# Comparing the advantages, disadvantages and diagnostic power of different non-invasive pre-implantation genetic testing: A literature review

**DOI:** 10.18502/ijrm.v22i3.16161

**Published:** 2024-05-15

**Authors:** Noorodin Karami, Farzaneh Iravani, Sareh Bakhshandeh Bavarsad, Samira Asadollahi, Seyed Mehdi Hoseini, Fateme Montazeri, Seyed Mehdi Kalantar

**Affiliations:** ^1^Abortion Research Centre, Yazd Reproductive Sciences Institute, Shahid Sadoughi University of Medical Sciences, Yazd, Iran.; ^2^Department of Genetics, Shahid Sadoughi University of Medical Sciences, Yazd, Iran.; ^3^Research Center for Food Hygiene and Safety, Shahid Sadoughi University of Medical Sciences, Yazd, Iran.; ^4^Biotechnology Research Centre, Yazd Reproductive Sciences Institute, Shahid Sadoughi University of Medical Science, Yazd, Iran.

**Keywords:** Spent culture media, Non-invasive pre-implantation genetic testing, Biopsy methods, Cell-free embryonic DNA.

## Abstract

To improve embryo transfer success and increase the chances of live birth in assisted reproductive methods, there is a growing demand for the use of pre-implantation genetic testing (PGT). However, the invasive approaches used in PGT have led to in vitrofertilization failure and abortions, increasing anxiety levels for parents. To address this, non-invasive PGT methods have been introduced, such as the detection of DNA in blastocoel fluid of blastocysts and spent culture media (SCM). These methods have proven to be minimally invasive and effective in detecting aneuploidy in the chromosomes of human embryos. This review aims to explore the different approaches to pre-implantation diagnosis, including invasive and non-invasive methods, with a particular focus on non-invasive PGT (niPGT). The search strategy involved gathering data from scientific databases such as PubMed, Google Scholar, and Science Direct using relevant keywords. The search was conducted until January 2023. In total, 22 studies have successfully reported the detection and amplification of cell-free DNA in the embryonic SCM. It is important to note that niPGT has some limitations, which include differences in indicators such as cell-free DNA amplification rate, concordance, level of maternal DNA contamination, sensitivity, and specificity between SCM samples and biopsied cells. Therefore, more extensive and detailed research is needed to fully understand niPGT's potential for clinical applications.

## 1. Introduction 

Pre-implantation genetic testing (PGT) gives us information about the genetic health of the embryo and has garnered more attention over the past 2 centuries. The development of safe and accurate diagnostic methods is the predominant key to achieving a healthy birth (1, 2). A wide range of new methods have been used in this field, which will be explained in the following sections. They can be used as assisted reproductive methods to help patients with infertility and repeated miscarriages to have a healthy baby. The prevalence of infertility varies in different countries and is estimated at approximately 9% and the rate of clinical abortions is estimated to be about 10–15% of all diagnosed pregnancies. Also, one of the causes of miscarriage and infertility is the increase and decrease in the number of chromosomes, which is called aneuploidy (3).

**Figure 1:**
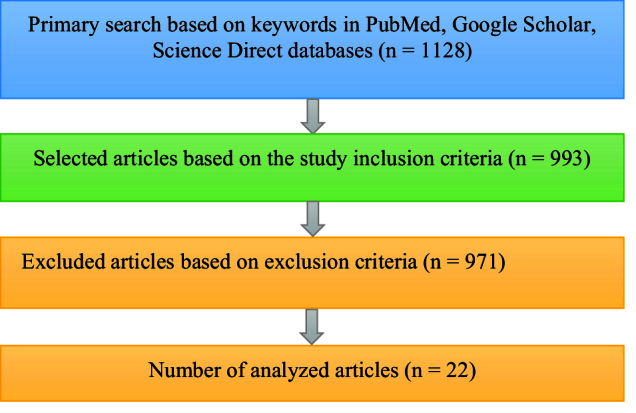
Flowchart of the methodology used in our study.

Therefore, PGT is one of the diagnostic techniques in the field of assisted reproductive technologies that can help increase the pregnancy rate in these patients. This study aims to introduce PGT, its types, and applications, focusing on non-invasive techniques that have received special attention recently. The data collection process in scientific databases such as PubMed, Google Scholar, and Science Direct using selected keywords started in February 2022 and continued until January 2023. The keywords included the following: invasive and non-invasive PGT, pre-implantation genetic diagnosis, PGT of aneuploidy (PGT-A), blastocyst biopsy methods, cell-free DNA (cfDNA), spent culture media/medium, and assisted reproduction. We found 1128 articles and analyzed 22 original articles evaluating the niPGT method, published in English until January 2023 (Figure 1). Animal studies, case reports, and review articles were excluded from the results.

## 2. Overview of PGT and its approaches

**Figure 2:**
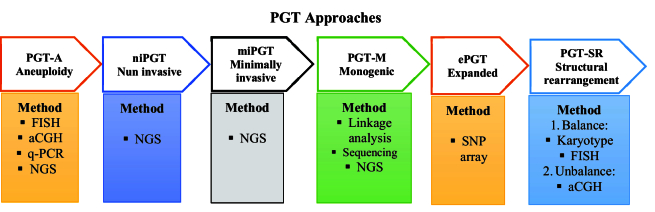
Overview of different PGT technical approaches. PGT: Pre-implantation genetic testing, PGT-A: PGT of aneuploidy, FISH: Fluorescence in situ hybridization, aCGH: Array comparative genomic hybridization, q-PCR: Quantitative-polymerase chain reaction, NGS: Next-generation sequencing, SNP: Single nucleotide polymorphism, PGD: Pre-implantation genetic, niPGT: Non-invasive pre-implantation genetic testing, miPGT: Minimally invasive pre-implantation genetic testing, PGT-M: PGT for monogenic disorders, ePGT: Expanded pre-implantation genetic testing, PGT-SR: Pre-implantation genetic testing for structural rearrangement.

The successful rate of in vitro fertilization (IVF) depends on multiple factors including the genetic complement of the embryo. Pre-implantation genetic screening (PGS) and genetic evaluation of the embryo with the use of the fluorescence in situ hybridization technique have been applied in IVF, since 1995 (4). Across these years, PGS have utilized several cytogenetics molecular techniques like comparative genomic hybridization, array-comparative genomic hybridization, single-nucleotide polymorphism array, quantitative-polymerase chain reaction (q-PCR), and next-generation sequencing. Although, it has some drawbacks it can improve pregnancy rates by the election of euploid embryos for transfer (5, 6). PGT (PGD) refers to the detection of known mutations or chromosomal rearrangements in embryos when one or both parents are carriers (7). Hence, it is clinically important to prevent the transmission of inherited disorders in the family to the future offspring (8). Since 2017, PGS and PGD were re-termed as PGT (9), and it is widely used in IVF centers around the world to select euploid embryos (Figure 2) (10).

PGT-A is a technique that is used to screen the numerical chromosome abnormalities in the embryo. It is used instead of PGS, comprehensive chromosome screening, and aneuploidy screening to identify euploid chromosomes during IVF (11). PGT for single gene/monogenic disorders (PGT-M), formerly known as PGD, is an early genetic diagnosis test for a single defective gene that can be inherited from a parent in a carrier family or formed during germ-line development (12–14). A balanced structural rearrangement is a type of chromosomal structural variant involving translocations, insertions, Robertsonian, and inversions of chromosomes without cytogenetically apparent gain or loss. Although the carriers are not phenotypically affected, it can impact meiotic segregation leading to unbalanced gametes and an increased risk of miscarriage. PGT for structural rearrangement can improve the reproductive product in affected couples and reduce the time to achieve a successful live birth (15–17). Expanded PGT combines polygenic risk score algorithms with the newest molecular biology techniques statutes (18–20).

Minimally invasive PGT uses cell-free embryonic DNA in spent culture medium (SCM) combined with blastocoel fluid (BF) to increase the amount of assayable cell-free embryonic nuclear DNA. Due to the aforementioned requirements, a new approach has recently been presented that is an alternative to TE biopsy and has attracted a lot of attention (21). This method, called non-invasive PGT (niPGT), examines the cfDNA released by an embryo into its adjacent culture medium (22). The clinical application of niPGT is based on the elucidation of cfDNA origin and the representation degree of the whole embryo (23). However, the cfDNA collection requires less skill and makes a lower risk to embryos because trophectoderm (TE) biopsy could affect embryo health and its potential implantation rate. Different studies have reported various concordance rates between TE and SCM samples. Thus, the accuracy, specificity, and sensitivity of niPGT must be confirmed in the larger clinical trials (11), and further investigations are needed to find out the accurate mechanisms underlying the release of embryonic DNA also the whole clinical potential of niPGT remains unknown and needs more discovery (24).

## 3. Types of biopsy methods for PGT

It is fair to say that PGT methods based on biopsies of polar bodies, blastomeres, or TE cells have been somewhat successful (25). Although isolation of the first polar body (PB) for PGT purposes is often considered less invasive than other methods (26). The most common form of biopsy in PGT comprised removing one or 2 blastomeres during the cleavage stage, which was frequently done on day 3 when the embryo typically had 6–10 cells. However, this strategy has several drawbacks including the presence of chromosomal mosaicism and the phenomenon of allele dropout (27). Also, PGT-A based on TE biopsy is currently the most widely used genetic test identifying de novo aneuploidy in embryos in clinical IVF.

The removal of 5–10 TE cells in the blastocyst biopsy usually does not affect the inner cell mass (ICM) cells; therefore, it is safer than other methods and is gradually replacing them (28). There are 3 main challenges of PGT associated with TE biopsy samples: 1) laborious and time-consuming biopsy procedure, 2) invasiveness, 3) subject to sampling bias -TE biopsy may not accurately represent the ICM and the rest of the TE. The initial report about the discovery of suitable DNA for amplification and genetic testing in the BF has aroused great curiosity among scientists. This discovery has opened up the possibility of a new era of minimally invasive PGT (29).

## 4. The necessity of using non-invasive methods in PGT

Although embryo biopsy is presently the method of choice over the world, it has significant drawbacks as well. First, an embryo biopsy can only be done during a particular stage of the embryo's growth. Because blastomere removal impacts developing embryos, day 3 (D3) cleaved embryos with fewer than 6 blastomeres are not candidates for biopsy. Similarly, to the quantity of biopsied cells has a detrimental impact on the implantation ability of blastocysts with inadequate TE quality. Secondly, while performing an embryo biopsy, only a small portion of the entire embryo is removed, which increases the risk of genetic misdiagnosis (false positives or negatives) in cases of embryo mosaicism. Third, invasive operations reduce an embryo's ability to reproduce. Embryo growth is less than ideal when a biopsy is performed at the cleavage stage. Because TE biopsy needs in vitro culture up until the blastocyst stage, there are worries related to its safety. Fourth, time- and money-consuming intrusive procedures are required. These difficult approaches call for advanced technical abilities, ongoing training, and suitable tools (such as laser equipment) (19, 24, 29, 30).

## 5. Origin, importance, and analytical workflow of cfDNA in SCM

Recently, studies have shown that DNA released by the embryo into the culture medium can be used for genetic testing as a noninvasive method (31). There are different views on the question regarding what the origin of cfDNA is. In general, it is believed that processes such as apoptosis, cell lysis, cell debris, or other mechanisms during embryonic development in embryo culture may cause the DNA of cells to break and form cfDNA fragments (32). Investigations revealed 2 types of DNA fragments: one between 160–220 bp and the other between 300–400 bp (33). Another study has reported apoptosis in ICM and TE between euploid cells and aneuploid cells in mouse models, and in aneuploid embryos, the percentage of apoptotic cells in ICM has been observed to be higher than in TE. Therefore, cfDNA is mainly formed through apoptosis and originates mainly from the ICM. However, more detailed and extensive studies on the origin of this type of DNA are still needed (34).

**Figure 3:**
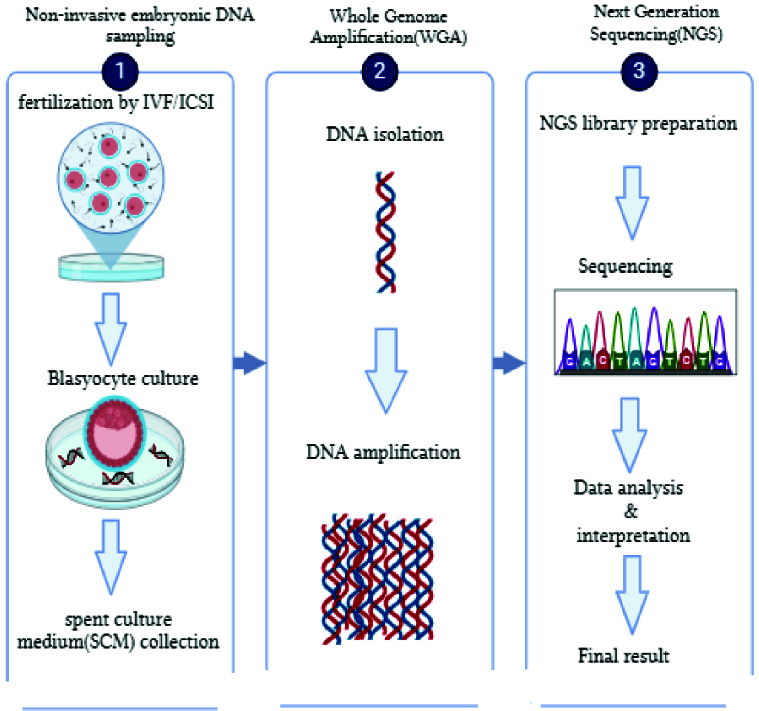
The workflow for analysis of embryonic cfDNA in blastocyst culture medium. ICSI: Intracytoplasmic sperm injection, IVF: In vitro fertilization. (The image was designed using BioRender.com database).

The recent finding of cfDNA in biological fluids has unlocked new perspectives for advancing non-invasive examinations within reproductive medicine. Indeed, researchers have identified cfDNA in the BF and used culture medium of embryos undergoing IVF procedures (18). Developing research has revealed the existence of cfDNA in various bodily fluids, blastocyst fluid, and the medium utilized to culture in vitro embryos (Figure 3). These findings have opened new ways for incorporating non-invasive methodologies into assisted reproductive technology. The utilization of cfDNA, which can be detected by the embryonic developmental culture material known as SCM, appears to be the most promising option for non-invasive PGT. A multitude of research groups have identified cfDNA and are currently undergoing assessment as a method for evaluating the chromosomal status of in vitro cultured embryos. A recent evaluation of 15 published studies has demonstrated that the detection of DNA in a SCM is a safe and efficacious approach for determining the chromosomal status of developing embryos. Nevertheless, the diverse methodologies employed in distinct studies have the potential to compromise the validity of the results, making it implausible to directly correlate the findings (18, 35–37).

## 6. Current studies on niPGT and their summary

**Figure 4:**
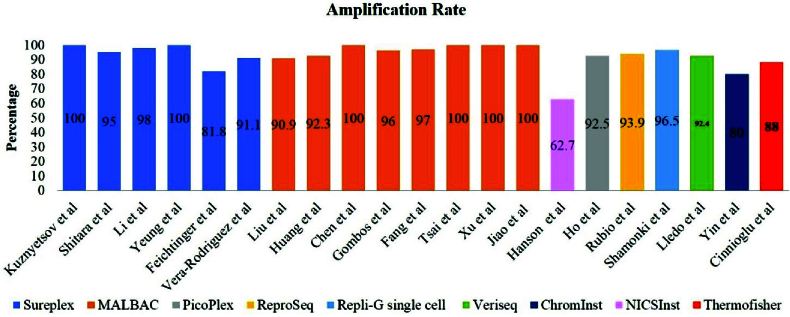
The rate of successful amplification of niPGT studies by various WGA methods. MALBAC: Multiple annealing and looping-based amplification cycle, NICS: Noninvasive implantation capability screening.

PGF for aneuploidy abnormalities is an increasingly used approach in helping couples with fertility-related problems. At present, the main drawback of the PGT-A method is its invasiveness, because it can lead to irreparable risks to the embryo (38). Therefore, there is an increasing need to develop non-invasive or minimally invasive approaches such as niPGT-A and minimally invasive PGT-A for clinical practice. Moreover, the successful amplification of the *TSPY1* gene on the Y chromosome in the culture medium to determine fetal sex led to the development of non-invasive pre-implantation approaches to the management of sex-linked diseases (39). A subsequent report demonstrated successful detection of the alpha thalassemia gene using quantitative-polymerase chain reaction. Interestingly, their finding showed that the detection rate obtained from SCM was higher than that in the embryo biopsy (88.6% and 82.1%, respectively) (40). Other studies used next-generation sequencing-based techniques to investigate cfDNA presented in the culture medium (Figure 4) (41, 42). Another research team achieved 100% successful amplification rate by using the noninvasive multiple annealing and looping-based amplification cycle (MALBAC)-NGS protocol on 42 SCM samples between days 3 and 5 (22).

Also, 100% successful DNA isolation rate was reported for 47 samples analyzed through a combined protocol of blastocyst culture medium and BF. After studying 166 SCM samples, a DNA amplification failure rate of 37.3% was observed. They concluded that the current form of niPGT-A makes its clinical application practically impossible (43). However, it seems that one of the weaknesses of their study was the low sample size and the use of a special amplification method called noninvasive chromosome screening inst library preparation kit Genomics. In a recent multicenter study with a high sample size, the amplification rate in embryonic culture medium samples was evaluated around 97.4% (1267/1301) (44, 45). Consequently, one of the reasons for the apparent difference between the successful rates of DNA amplification in these studies may be due to differences in the study design.

**Figure 5:**
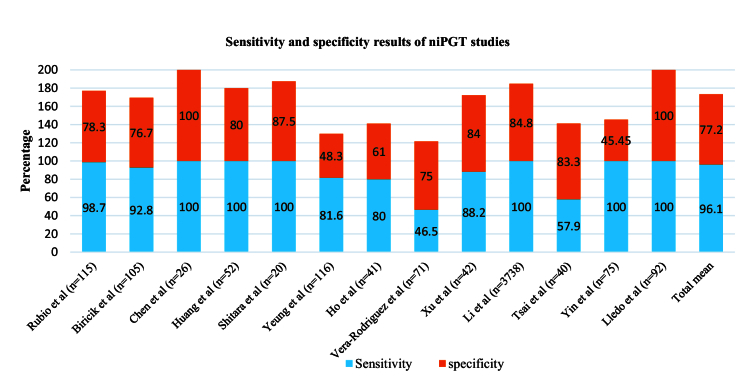
Sensitivity and specificity of results in niPGT studies.

Also, in studies related to niPGT, different levels of sensitivity and specificity have been reported (Figure 5). The highest level of sensitivity, even up to 100%, was observed in some studies, and the highest rate of specificity has been reported in the study of other researches (8, 46). One of the reasons for the high sensitivity and specificity of these studies can be related to the type of whole genome amplification (WGA) method used for DNA amplification and culture time; most of these studies have chosen MALBAC or SurePlex methods and D5/D6 culture time (47). Additionally, to obtain the concordance rate, it is necessary to compare niPGT results with embryo biopsy results. Studies have used different biopsy methods for this comparison, including PB biopsy, TE biopsy (16, 31, 48, 49), BF (50, 51), and whole embryo (28). For the PB biopsy, a lower concordance rate was obtained (27%), while for the BF 87.5% rate was observed. Also, when TE biopsy was used, the concordance rate was between 3.5–93.8% and these differences were due to other reasons, such as the type of cfDNA amplification method, the WGA method used, the type of culture medium and its volume, the level of maternal contamination, etc. can happen, which will be discussed further (52).

## 7. Tips and tricks in niPGT using SCM

Our analysis of various studies led to the identification of important tips and tricks in niPGT using SCM, which are summarized below:

### Types of embryo culture medium systems 

Embryo culture systems are commercially divided into 2 types: single-step and 2-step culture media. 2-step or sequential culture medium, takes into account the needs of the embryo in its different stages of growth and development, its essential nutrients are provided in the culture medium. In single-step culture medium, the nutritional requirements of the embryo were mixed and added to the culture medium in one step. The embryos then chose the nutrients based on their needs. The type of culture medium is important in 2 aspects: 1) Degradation of cfDNA in culture medium decreases the DNA quality, as a result, the amount of time spent on cfDNA degradation can be diminished by changing the culture medium. 2) By changing the culture medium, the amount of maternal DNA contamination produced by cumulus cells can be reduced. In our investigations, it was found that most of the studies used a 2-step system to analyze the SCM of the embryos (Table I). In some studies, however, no significant difference was observed, by comparing both types of culture medium system regarding their effect on the accuracy of niPGT-A results (44).

**Table 1:** Summary of studies evaluating the amplification and analysis outcomes of cell-free DNA in the SCM


**Author, yr (Ref)**	**Number of samples**	**Amplification rate**	**Concordance rate**	**Discordance rate**	**Culture volume (µL)**	**Culture medium (single/2-stage)**	**Fertilization method**	**Manipulation of embryo**	**Culture time**	**Amplification method**	**WGA method**
**Kuznyetsov ** ***et al.*** **, 2020 (29)**	47	100 (47/47)	97.8 (45/47)	2.2 (2/47)	25	2 steps	ICSI	Cryopreservation on D4	D4/D5/D6	SurePlex (Illumina)	NGS
**Chen ** ***et al.*** **, 2022 (53)**	26	100 (26/26)	80.8 (21/26)	19.2 (5/26)	10	2 steps	ICSI	Cryopreservation	D5/D6/D7	MALBAC (Yikon)	NGS
**Gombos ** ***et al.*** **, 2021 (35)**	753	95–96	- -	20–40	2 steps	ICSI	Assisted hatching on D3	D3	MALBAC (Yikon)	NGS
**Huang ** ***et al.*** **, 2022 (51)**	166	62.7 (104/166)	59.6 (62/104)	40.4 (42/104)	30	2 steps	IVF and ICSI	Cryopreservation D3	D5/D6/D7	NICSInst Genomics (Yikon)	NGS
**Shitara ** ***et al.*** **, 2021 (31)**	20	95 (19/20)	56.3 (9/16)	43.7 (7/16)	- 2 steps	IVF	Cryopreservation on D5/D6	D5/D6	SurePlex (Illumina)	NGS
**Brouillet ** ***et al.*** **, 2020 (54)**	3738	98 (3662/3738)	87.2	12.8	30	- ICSI	Cryopreservation on D3	D3-D5/D6	SurePlex (BlueGnome)	NGS
**Yeung ** ***et al.*** **, 2019 (52)**	168	100 (26/26)	87.8 (21/26)	19.2 (5/26)	10	2 steps	ICSI	Assisted hatching on D3	D3-D5/D6	SurePlex (Illumina)	NGS
**Tsai ** ***et al.*** **, 2022 (55)**	40	100 (40/40)	67.7 (21/31)	32.3 (10/31)	30	Single step	IVF/ICSI	Cryopreservation	D3-D5/D6	MALBAC (Yikon)	NGS
**Alteri ** ***et al.*** **, 2023 (28)**	57	96.5 (55/57)	33.3 (2/6)	- 15	2 steps	ICSI	Assisted hatching on D3	D3-D5/D6	Repli-G single-cell kit (Qiagen)	aCGH
**Xu ** ***et al.*** **, 2016 (56)**	42	100 (42/42)	85.7 (36/42)	14.3 (6/42)	30	2 steps	ICSI	Cryopreservation on D3	D3-D5	MALBAC (Yikon)	NGS
**Jiao ** ***et al.*** **, 2019 (30)**	62	100 (62/62)	76.19 (16/21)	- 12	- ICSI	Freeze-thaw. Laser collapse	D5/D6	MALBAC (Yikon)	NGS
**Feichtinger ** ***et al.*** **, 2017 (57)**	22	81.8 (55/57)	72.2 (13/18)	27.8 (5/18)	25	Single step	ICSI	Cryopreservation on D3	D1-D5/D6	SurePlex (Illumina)	aCGH
**Vera-Rodriguez ** ***et al.*** **, 2017 (58)**	56	91.1 (51/56)	33.3 (17/51)	- 20	2 steps	ICSI	Assisted hatching on D3	D3-D5	SurePlex + ReproSeq (Illumina)	NGS
**Rubio ** ***et al.*** **, 2020 (44)**	1301	97.5 (79/80)	78.2 (866/1108)	137 false positive, 92 false negative	10	A single step or 2 steps	IVF/ICSI	Cryopreservation + cryopreservation on D3	D6/D7	- NGS
**Liu ** ***et al.*** **, 2020 (59)**	88	90.9 (80/88)	64.52	35.48	30	Single step	ICSI	Cryopreservation	D1-D5	MALBAC (Yikon)	NGS
**Ho ** ***et al.*** **, 2018 (60)**	41	92.5 (37/40)	65 (26/40)	8 false positive, 3 false negative	25	Single step	ICSI	Cryopreservation + assisted hatching on D3	D3-D5	PicoPlex (Rubicon)	NGS
**Rubio ** ***et al.*** **, 2019 (47)**	115	93.9 (108/115)	Overall: 7/78 (108/85) D5: 63 (27/17) D6/7: 84 (81/68)	8 false positive, 1 false negative	10	Single step	IVF	- D4-D5/D6/D7	ReproSeq (Life Technologies)	NGS
**Xu ** ***et al.*** **, 2016 (50)**	170	97	- -	20–25	2 steps	ICSI	Cryopreservation	D3-D5/D6	MALBAC (Yikon)	NGS
**Yin ** ***et al.*** **, 2021 (48)**	75	80 (60/75)	89.83 (53/59)	10.17 (6/59)	25	2 steps	IVF	Cryopreservation	D5	ChromInst (Yikon)	NGS
**Lledo ** ***et al.*** **, 2018 (37)**	92	92.4 (85/92)	74.6 (62/83)	25.4 (21/83)	20	2 steps	IVF	Assisted hatching on D3	D5-D6	SurePlex	NGS
**Huang ** ***et al.*** **, 2022 (51)**	52	92.3 (48/52)	93.8 (45/48)	6.2 (3/48)	25	Single step	ICSI	Assisted hatching on D3	D4-D5/D6/D7	Modified MALBAC (Yikon)	NGS
**Cinnioglu ** ***et al.*** **, 2023 (61)**	50	88 (44/50)	78.9 (30/38)	21 (8/38)	30	- -	- D5-D6	Thermofisher	NGS
SCM: Spent culture medium, WGA: Whole-genome amplification, ICSI: Intracytoplasmic sperm injection, D: Days, NGS: Next-generation sequence, IVF: In vitro fertilization, aCGH: Array comparative genomic hybridization, MALBAC: Multiple annealing and looping-based amplification cycle, NICSInst: Noninvasive chromosome screening inst library preparation kit											

### The volume of embryo culture medium

In general, studies on the amount of SCM collected to amplify DNA present in culture media have reported a wide range. This factor is important, because it potentially has a direct effect on the proportion of extra-embryonic DNA collected and changes the initial concentration of the template. In the niPGT method, determining the optimal volume for embryo culture and subsequent analysis is challenging and problematic. These challenges include:

1. Inhibitory effect of culture medium components on DNA amplification during WGA and PCR.

2. Design of the most commercial WGA protocols with very small volumes (usually < 10 µL) for use in SCM. Using more volume leads to increased costs. To overcome these challenges, some authors have suggested embryo culture in smaller volumes (47). This reduction in culture volume requires careful and detailed examinations because it is vital to ensure normal growth and survival and no risk to the embryo.

### Timing of SCM collection

**Figure 6:**
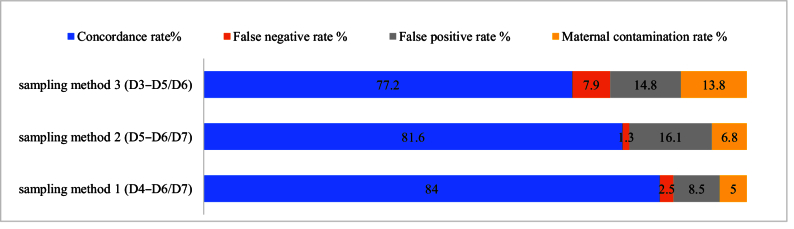
Comparison of different sampling methods and their results in a study by Huang et al. (51).

The timing of SCM collection during embryo culture is also considered one of the crucial factors that may lead to different results of the analysis of niPGT. In a study by Lane et al., they observed that by comparing the time of collecting the medium, a higher accuracy for SCM samples D4–D5 compared to D3–D5 was obtained (62). Also, another study reported SCM consistency and decreased levels of maternal contamination of D4–D6/7 significantly higher than D4–D5. In figure 6, the results for concordance rate, false positive and negative rate, and maternal DNA contamination are shown for different sampling methods (63). Sampling method 1, which was performed by collecting SCM on D4–D6/7, showed the highest concordance rate (84%), the lowest false positive rate, and maternal contamination (8.5% and 5%, respectively).

### Methods of cfDNA amplification

There are several types of WGA techniques used to amplify cfDNA in SCM, including multiple annealing and looping amplification cycles (MALBAC) and sureplex/picoplex, repli-G single cell, multiple displacement amplification, and veriseq. As shown in figure 4, several niPGT-A studies have used the MALBAC method to amplify cfDNA in SCM with a successful rate of over 90%, and using the PicoPlex/SurePlex method, a successful amplification rate of over 80% has been reported. Therefore, using the aforementioned methods for cfDNA amplification in SCM is more common and practical. Also, the high application of the MALBAC method for cfDNA analysis can be due to its high genome coverage and very low allele deletion ratio (64).

### Limitations of performing niPGT

Recent developments in the field of cfDNA discovery in BF and SCM have given rise to a greater interest in the niPGT field to make a less invasive medical diagnosis of embryo health status, but it has some limitations. One of the biggest challenges of niPGT is a lower quantity and lesser quality of the cell-free genetic material, and its unspecified origin (65). But some researchers believe that despite the fact that embryonic and maternal DNA differences are still ineffectively characterized, this problem does not make a threat to diagnostic validity of niPGT for monogenic and X-linked disease. In these cases, the only limitation might be the incomplete representation of the whole embryonic genome by cfDNA (21). Hence, low abundance and poor integrity, make technical challenges for genetic analysis as in this time it is not clear which method is more accurate to analyze the extra-embryonic DNA and to get a precise clinical diagnosis, the origin of extra-embryonic DNA should be definite and the factors that create discordance with results must be discovered.

## 8. Conclusion

In this study, we investigated the PGT approach and its types, especially the non-invasive methods, and explained the factors affecting the rate of amplification, concordance, sensitivity, specificity, and current limitations in the field of using cfDNA in PGT. Much attention has been paid to non-invasive PGT due to its cost-effectiveness, no damage to the fetus, and no need for biopsy. However, important challenges in its clinical application include differences in results obtained from niPGT compared to results obtained from biopsy samples, maternal DNA contamination, fetal mosaicism, and the relatively low abundance of DNA present in SCM. Therefore, it seems necessary to conduct more research to evaluate the potential of using PGT in clinical practice.

##  Data availability

Not applicable.

##  Author contributions

SM Kalantar, F Montazeri, and N Karami designed the study and conducted the paper.

N Karami, F Iravani and F Montazeri had full access to all the data in the study and takes responsibility for the integrity of the data and the accuracy of presented data.

F Iravani, S Bakhshandeh Bavarsad, SM Hoseini and S Asadollahi: Drafting of the manuscript.

SM Hoseini, S Asadollahi, and S Bakhshandeh Bavarsad reviewed the article and made the necessary corrections as requested by the referees.

##  Conflict of Interest

The authors declare that there is no conflict of interest.
